# The estrogen receptor coactivator AIB1 is a new putative prognostic biomarker in ER-positive/HER2-negative invasive lobular carcinoma of the breast

**DOI:** 10.1007/s10549-019-05138-7

**Published:** 2019-02-22

**Authors:** Ulrik Narbe, Martin Sjöström, Carina Forsare, Pär-Ola Bendahl, Sara Alkner, L. M. Fredrik Leeb-Lundberg, Kristina Lövgren, Lisa Rydén, Christian Ingvar, Mårten Fernö

**Affiliations:** 1 Department of Clinical Sciences, Division of Oncology and Pathology, Lund University, Medicon Village, 223 81 Lund, Sweden; 2 Department of Clinical Sciences, Division of Surgery, Lund University, Lund, Sweden; 3 Skåne Clinic of Oncology, Skåne University Hospital, Lund, Sweden; 4 Department of Oncology, Växjö Central Hospital, Växjö, Sweden; 5 Department of Surgery, Skåne University Hospital, Lund, Sweden; 6 Department of Experimental Medical Science, Lund University, Lund, Sweden

**Keywords:** Invasive lobular carcinoma (ILC), Amplified in breast cancer 1 (AIB1), G protein-coupled estrogen receptor (GPER), Androgen receptor (AR), Prognostic biomarkers, Gene expression

## Abstract

**Purpose:**

According to the 2017 St Gallen surrogate definitions of the intrinsic subtypes, Ki67, progesterone receptor (PR) and Nottingham histological grade (NHG) are used for prognostic classification of estrogen receptor (ER) positive/HER2-negative breast cancer into luminal A- or luminal B-like. The aim of the present study was to investigate if additional biomarkers, related to endocrine signaling pathways, e.g., amplified in breast cancer 1 (AIB1), androgen receptor (AR), and G protein-coupled estrogen receptor (GPER), can provide complementary prognostic information in a subset of ER-positive/HER-negative invasive lobular carcinoma (ILC).

**Methods:**

Biomarkers from 224 patients were analyzed immunohistochemically on tissue microarray. The primary endpoint was breast cancer mortality (BCM), analyzed with 10- and 25-year follow-up (FU). In addition, the prognostic value of gene expression data for these biomarkers was analyzed in three publicly available ILC datasets.

**Results:**

AIB1 (high vs. low) was associated to BCM in multivariable analysis (adjusted for age, tumor size, nodal status, NHG, Ki67, luminal-like classification, and adjuvant systemic therapy) with 10-year FU (HR 6.8, 95% CI 2.3–20, *P* = 0.001) and 25-year FU (HR 3.0, 95% CI 1.1–7.8, *P* = 0.03). The evidence of a prognostic effect of AIB1 could be confirmed by linking gene expression data to outcome in independent publicly available ILC datasets. AR and GPER were neither associated to BCM with 10-year nor with 25-year FU (*P* > 0.33). Furthermore, Ki67 and NHG were prognostic for BCM at both 10-year and 25-year FU, whereas PR was not.

**Conclusions:**

AIB1 is a new putative prognostic biomarker in ER-positive/HER2-negative ILC.

**Electronic supplementary material:**

The online version of this article (10.1007/s10549-019-05138-7) contains supplementary material, which is available to authorized users.

## Introduction

Estrogen receptor (ER) positive/HER2-negative breast cancer (BC) comprises 75–80% of all BC and this fraction is even higher (> 90%) in invasive lobular carcinoma (ILC) [1, 2]. ILC is the second most common histological type of BC after invasive carcinoma of no special type (NST) and comprises approximately 10% of all invasive BC [3]. ILC has, compared to NST, distinct clinicopathological [4–7] and genomic features [8–10], and the response to adjuvant systemic therapy differs [11, 12]. ILC also has a higher incidence of late recurrences but the overall prognosis seems to be the same [8, 12, 13]. In spite of the differences, current surgical and adjuvant treatments are similar. According to the 2017 St Gallen surrogate definitions of the intrinsic subtypes, proliferative fraction (Ki67), progesterone receptor (PR) status, and Nottingham histological grade (NHG) are used to classify ER-positive/HER2-negative BC, as luminal A- or luminal B-like [14]. The luminal-like (HER2-negative) classification together with tumor size, axillary lymph node status (nodal status), and age is widely used in the clinic for prognostication and treatment decisions (endocrine therapy ± chemotherapy). Nevertheless, these established prognostic variables still have their limitations, and there is an unmet need for additional prognostic biomarkers.

The androgen receptor (AR) belongs to the steroid nuclear receptor family and is frequently expressed in BC, especially in ER-positive ILC (> 85%) [12, 15, 16]. The prognostic role of AR in BC is still unclear with some studies showing that AR positivity is associated with better prognosis [17–19] and others showing non-prognostic results [20, 21]. The prognostic impact of AR in ILC is sparsely studied. Amplified in breast cancer 1 (AIB1) is a member of the steroid receptor coactivator family and interacts with ER. AIB1 is often expressed in BC and high AIB1 is implicated to be a negative prognostic factor and at the same time a predictive factor for response to endocrine therapy, although the findings are not unanimous [22–28]. G protein-coupled estrogen receptor (GPER) is distinct from ER and mediates non-genomic estrogenic responses. The reported prognostic value of GPER expression in BC is inconsistent [29–33]. Furthermore, lack of GPER in the plasma membrane (PM GPER-negativity) has been identified as a good prognostic feature in ER-positive BC [29]. To the best of our knowledge, no previous studies of either AIB1 or GPER as a prognostic factor exclusively in ILC has been carried out.

The aim of the present study was to investigate if these new putative prognostic biomarkers (AR, AIB1, and GPER), related to endocrine signaling pathways, can provide complementary information to established prognostic variables in a well-characterized case series of ER-positive/HER2-negative ILC with long-term follow-up (FU), and furthermore, if our immunohistochemical (IHC) findings could be validated in three independent publicly available gene expression ILC datasets [34–36].

## Patients and methods

### Study population

Between 1980 and 1991, 319 cases of female primary breast cancer were classified as ILC at the Departments of Pathology, Lund University Hospital, and Helsingborg Hospital, Sweden.

**Fig. 1:**
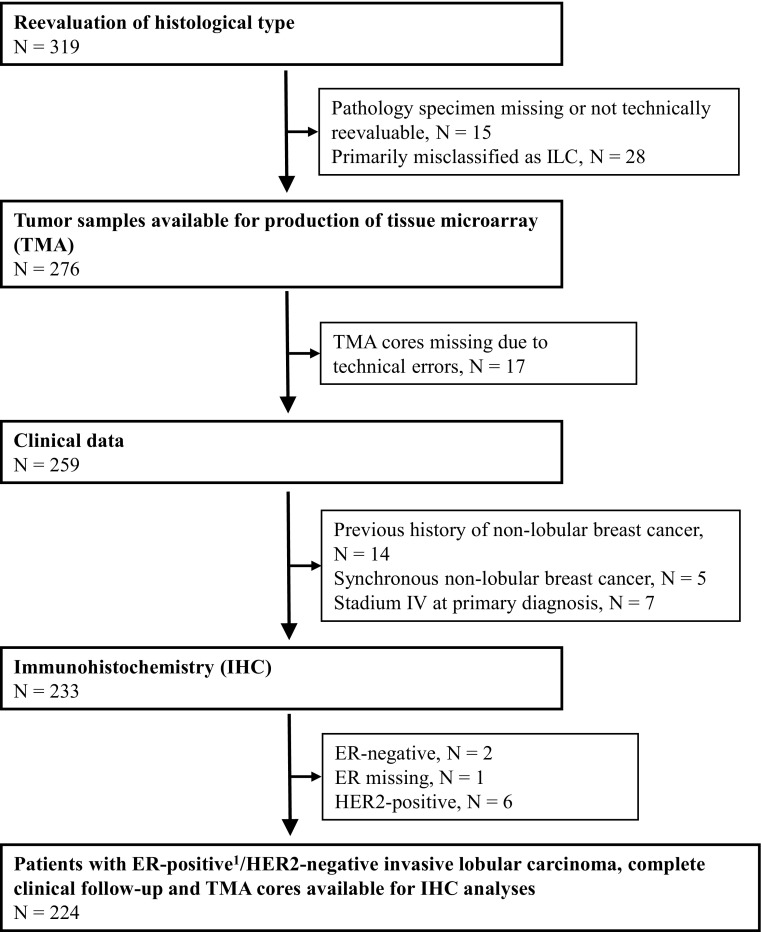
Consort diagram: Breast cancer patients with tumors primarily classified as invasive lobular carcinomas (ILC) at the Department of Pathology, Skåne University Hospital Lund and Helsingborg Hospital, (1980–1991), *N* = 319. ER positivity (≥ 1%) was confirmed with IHC staining on tissue microarray in *N* = 200 and whole tissue sections in *N* = 21, and with cytosol-based methods in *N* = 3 tumor samples

Reevaluation of histological type was performed for all tumors by two clinical pathologists, specialized in breast cancer, without knowledge of clinical data. In total, 95 patients were excluded leaving 224 patients available for further analyses in the present retrospective study (Fig. 1). A further subdivision of the ILCs was not performed. A minority (approximately 15%) had an involvement of minor non-lobular invasive foci. E-cadherin was analyzed with IHC (Clone NCH-38, M3612 DAKO/Agilent 1:100) and loss of E-cadherin expression was found in 85% of the included tumors. The study population is based on a previously reported cohort [37], with the addition of 52 ILC cases from Helsingborg Hospital. Furthermore, this study has a longer FU time and all included biomarkers were assessed by IHC on tissue microarray (TMA), compared to previous whole tissue section analyses of ER, PR, HER2, and Ki67. NHG was reevaluated on whole tissue sections according to Elston and Ellis [38]. Patient and tumor characteristics were retrieved from clinical records and pathology reports (Table 1), as were FU data.

**Table 1:** Patient and tumor characteristics (*N* = 224)

Variables	No (%)
Age	
Median, years (range)	62 (36–87)
Menopausal status	
Premenopausal	56 (26)
Postmenopausal	161 (74)
Unknown	7
Type of surgery	
BCS	49 (22)
BCS (no ALND)	4
BCS + ALND	45
Mastectomy	175 (78)
Mastectomy (no ALND)	7
Mastectomy + ALND	168
Tumor size (mm)	
pT1 (≤ 20)	125 (57)
pT2 (> 20 and ≤ 50)	83 (37)
pT3 (> 50)	13 (6)
Undefined	3
Nodal status	
pN-(ALND)	129 (61)
cN-(no ALND)^a^	11
pN+	84 (39)
pN1 (1–3)	40 (19)
pN2 (4–9)	33 (15)
pN3 (> 9)	11 (5)
Adjuvant therapy^b^	
None	77 (35)
RT (total)	110 (49)
CT (total)	5 (2)
ET (total)	91 (41)
RT (monotherapy)	50 (22)
CT (monotherapy)	3 (1)
ET (monotherapy)	33 (15)
RT + CT	2 (1)
RT + ET	58 (26)
CT + ET	0 (0)
RT + CT + ET	0 (0)
Unknown	1

*BCS* Breast-conserving surgery, *ALND* Axillary lymph node dissection, *c* clinical, *p* pathological, *N−* node negative, N+ node positive

^a^The main reason for not undergoing ALND was a clinical node-negative (cN-) status in patients with co-morbidity and high age at diagnosis

^b^Adjuvant therapy: *RT* radiotherapy, *CT* chemotherapy, *ET* endocrine therapy

### TMA preparation

TMAs were prepared from paraffin-embedded primary tumor blocks, using a manual arrayer (Beecher Instruments Inc.). Three cylindrical cores (triplets) with a diameter of 0.6 millimeter were taken from morphologically representative regions of the primary tumor blocks and transferred into a recipient paraffin block. Sections (3–4 µm) were taken from each TMA block and transferred to glass slides.

### IHC staining and scoring of prognostic biomarkers

**Fig. 2:**
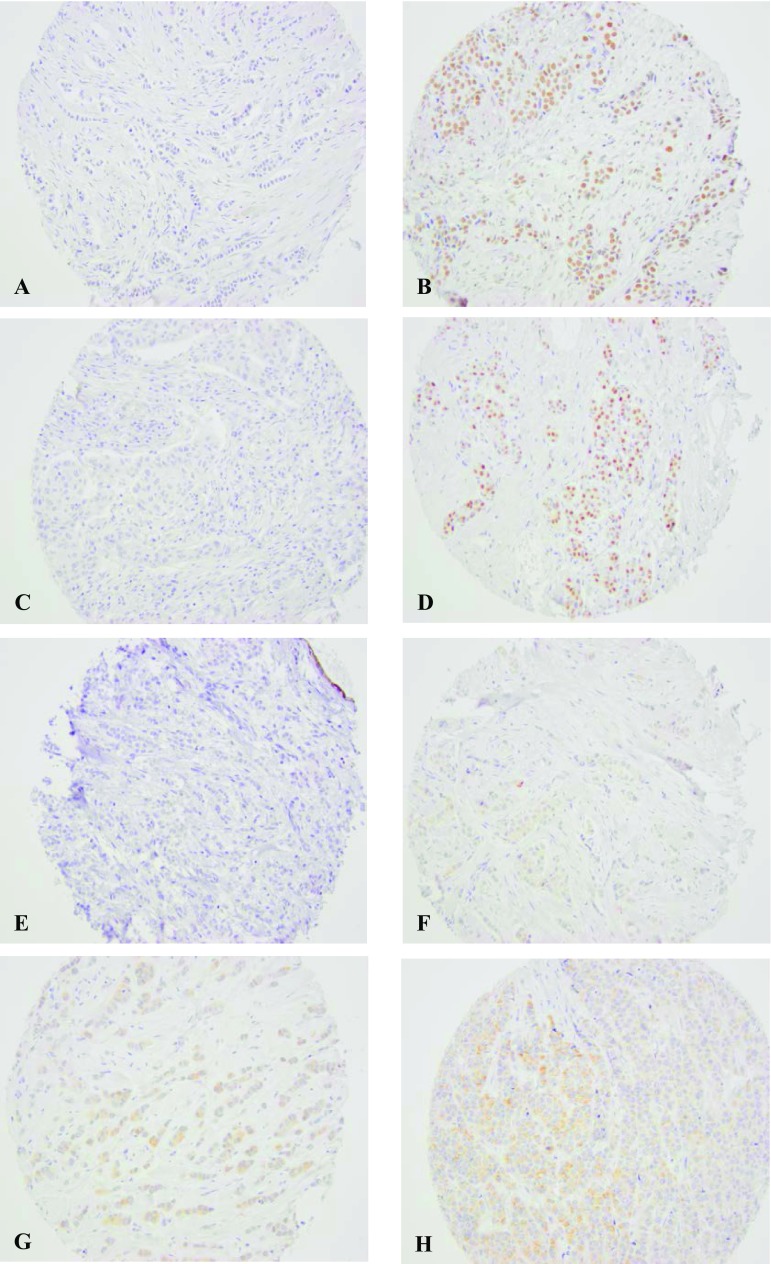
Representative images of immunohistochemical staining of AIB1, AR, and GPER: **a** AIB1 low (score 0) **b** AIB1: high (score 6) **c** AR negative (≤ 10%) **d** AR positive (> 10%). **e** Total GPER negative (level 0) **f** Total GPER very weak (level 1) **g** Total GPER weak (level 2) **h** Total GPER moderate (level 3). None of the TMAs were classified as total GPER strong (level 4)

IHC staining was carried out by an automatic immunostainer (TechMate^TM^500 Plus, DAKO), as previously described [39]. Each TMA section was digitally scanned and the images were evaluated using PathXL/Xplore (Philips). AIB1, AR, and GPER were assessed by two independent observers without knowledge of clinical data. Sections with less than 50 invasive cells were excluded. For the great majority (> 90%), more than 100 cells could be evaluated. Triplets of TMA cores from every tumor were assessed and in case of different staining scores between the cores, the highest score was chosen, except for GPER where the mean was used. Stains with discordant scoring between the observers were re-examined to reach consensus if the score differed by more than one step, otherwise the mean score was used. All cut-offs were decided according to a predefined protocol before linking protein expression to survival data. None of the biomarkers displayed any stromal staining. (Fig. 2).

For AIB1 detection, a monoclonal IgG antibody (Clone 34/AIB-1 1:40, BD Bioscience) was used. This antibody has a confirmed specificity [40] and has been used in several previous clinical studies [39–41]. AIB1 expression was analyzed in line with previous publications [27, 28, 39]. Each sample was semi-quantitatively scored from 0 to 3 for percentage of stained nuclei and staining intensity. IHC staining was exclusively seen in the nucleus. Proportion score 0 represented no stained nuclei, 1:1 to 10%, 2:11 to 50%, and 3:51 to 100%. Staining intensity 0 represented negative staining, 1 weak, 2 moderate, and 3 intense staining. Proportion and intensity scores were added to a total score ranging from 0 to 6. The total scores were categorized into three groups: 1 (score < 5), 2 (score 5 or 5.5^1^), and 3 (score 6). In line with results from the ER-positive/HER2-negative subgroup in a previous study from our group, the groups 1 and 2 were combined, resulting in two prognostic groups: high-AIB1 (score 6) and low-AIB1 (score < 6) [27].

AR expression was analyzed using AR-antibody M3562 (DAKO) 1:100. The percentage of stained nuclei was scored and a value > 10% was considered positive [17].

Staining of GPER was performed using the GPER-antibody AF 5534 (R&D System) 1:50 with confirmed specificity for GPER [30]. Total GPER staining was scored, according to a previous study from our group, as intensity at 5 levels (0 negative, 1 very weak, 2 weak, 3 moderate, and 4 strong) [29]. PM GPER staining was scored as the intensity at three levels (0 negative, 1 weak, and 2 strong). Level 1 to 2 were combined ending up with a binary variable (negative vs. positive).

ER (ER-alpha clone SP1, RM-9101 Thermo Scientific 1:200) and PR expression (Clone PgR636, M3569 DAKO 1:100) were analyzed with IHC and a score of ≥ 1% stained nuclei was considered positive. PR expression was also analyzed with a 20% cut-off value for the luminal-like classification.

Ki67 (Clone MIB-1, M7240 DAKO 1:200) proliferation index was considered high if ≥ 24% cells were stained. The cut-off value was set at this level to mimic the fraction of high Ki67 tumors (7.8%) in our previous whole tissue section analyses of ILC [37].

HER2 (CB11, Novocastra, 1:200) was categorized into four different IHC groups depending on the cell membrane staining intensity: 0, 1+, 2+, 3+. A value of IHC 3+ was considered as HER2 positive. A HER2 gene amplification test was not performed.

### Luminal-like classification

Based on the 2017 St Gallen luminal-like definitions [14], the tumors were divided into: luminal A-like (HER2-negative): grade 1 + 2, low Ki67, and PR > 20% and luminal B-like (HER2-negative): at least one of the three criteria fulfilled: grade 3, high Ki67, or PR ≤20%.

#### Description of gene expression datasets

In order to validate our IHC findings, we identified three independent publicly available gene expression ILC datasets [34–36].

The Metzger Filho et al. dataset [36] was downloaded from Gene Expression Omnibus (GEO) with accession number GSE88770, and was originally analyzed on the Affymetrix Human Genome U133 Plus 2.0 Array platform. It consists of 117 ILC tumors of which 100 where ER-positive/HER2-negative, and primary endpoint was distant disease-free survival (DDFS). Preprocessing of the data was made by the original authors.

The Michaut et al. [35] dataset was downloaded from GEO with accession number GSE68057, and data were originally obtained by an Agendia platform. It consists of 137 ILC tumors of which 108 where ER-positive/HER2-negative, and primary endpoint was recurrence-free survival (RFS). The file marked as “processed data based on older annotations” was used, and patient data were downloaded directly from the publication website. Preprocessing of the data was made by the original authors.

The METABRIC dataset [34] was downloaded from http://www.cBioPortal.org on 2018-01-15, and the datafile “data_expression” containing log2-transformed data was used. The original gene expression data were obtained by Illumina HT12 v3 microarray platform. The data were subset for ILC tumors. It consisted of 141 ILC tumors of which 123 where ER-positive/HER2-negative, and primary endpoint was overall survival (OS). Log intensity levels were used, as provided by the original authors.

No further preprocessing was done for the datasets, as we were interested in the relative gene expression levels within a cohort. Of note is the old annotations for some of the genes where GPER is also annotated as CMKRL2, and AIB1 is annotated as NCOA3. We have changed to a consistent use of gene symbols for clarity.

The cut-off values were set at levels to mimic the fractions of the concurrent IHC analyses in this study, and further statistical analysis was performed on the gene expression data in the same way as for the IHC data. [Distribution: 7% high AIB1, 93% AR positivity and total GPER: 0 (28%); 1 (42%); 2 (29%); 3 (1%); and 4 (0%)].

### Statistical analysis

Associations between AIB1, AR, GPER, and other prognostic factors were assessed using Pearson’s chi^2^ test. A trend version of this test, which is equivalent to a test for zero slope in a linear regression model, was used if one or both variables in a pair was ordinal with more than two categories (Table 3). The primary study endpoint was cumulative breast cancer mortality (BCM). For each patient, the FU time was counted from the date of surgery until death with or without breast cancer or, for the survivors, until June 2015. For the gene expression datasets, we used the same endpoints as originally published. The log-rank test was used to compare BCM, or other endpoints, in different strata (for variables with three or more ordered categories, a log-rank test for trend was used). Cause-specific Cox proportional hazards regression was used for estimation of hazard ratios (HR) which hence shall be interpreted as relative effects in a world where all other causes of death than breast cancer have been eliminated. Proportional hazard assumptions were checked graphically for each biomarker, and were found to be violated for, e.g., high vs. low AIB1 (Fig. 2). Hence, estimated HRs depend on FU time. Our pragmatic solution to this problem was to restrict the FU to the first 10 years. Complementary analyses with 25 years of FU were also performed to show how the estimated effects on BCM for the biomarkers level off with increased FU. These long-term effects should be cautiously interpreted as time averages. The FU exceeded 25 years for 42 patients, but since no breast cancer deaths occurred among them, this additional FU was not included when biomarker expression was analyzed in relation to BCM. All tests were two-sided and the corresponding unadjusted *P* values should be regarded as level of evidence against the null hypotheses tested. In the survival analyses, NHG, and nodal status were analyzed as factor variables on three levels, age as a continuous variable, and all other factors as dichotomous covariates. The statistical analysis software Stata version 15 (StataCorp, College Station, TX, USA) was used for statistical calculations.

The REMARK recommendations for reporting of tumor biomarker studies were followed [42].

## Results

### Follow-up data

Ninety-two patients (41%) had a diagnosed recurrence at last FU, and the distribution of site of first recurrence was as follows: local in 23 patients, regional in 7, and distant in 62. In addition, 23 (10%) patients had developed a contralateral BC. At the end of the study, 66 patients (29%) had died from breast cancer and 99 (44%) from other causes. The remaining 59 patients (26%) were still alive and had a median FU of 26 years (range 0.7–35 years) in June 2015.

### AIB1, AR, and GPER and their associations to other prognostic factors

Seven percent of the tumors were high-AIB1 (14/208) and 93% were AR positive (183/196). For total GPER, most tumors were negatively stained (level 0 28%) or showed very weak (level 1 42%) and weak (level 2 29%) staining intensity, whereas only three tumors had a moderate staining (level 3) and no tumor showed a strong staining (level 4) intensity. Based on these skewed total GPER distribution, we decided to combine level 2 to 4, resulting in analyses with three prognostic categories. Furthermore, with only four PM GPER-positive tumors, it was not meaningful to analyze this biomarker in relation to BCM (Table 2).

**Table 2:** Distribution of biomarkers and NHG

Variables	No (%)
PR	
Positive (≥ 1%)	162 (81)
Negative (< 1%)	37 (19)
Missing	25
Ki67	
Low (< 24%)	182 (92)
High (≥ 24%)	15 (8)
Missing	27
AIB1	
Low (score < 6)	194 (93)
High (score 6)	14 (7)
Missing	16
GPER	
Negative (0)	59 (28)
Very weak (1)	87 (42)
Weak (2)	60 (29)
Moderate (3)	3 (1)
Strong (4)	0 (0)
Missing	15
PM GPER	
Positive (1 + 2)	4 (2)
Negative (0)	205 (98)
Missing	15
AR	
Positive (> 10%)	183 (93)
Negative (≤ 10%)	13 (7)
Missing	28
NHG	
1	28 (14)
2	161 (80)
3	13 (6)
Missing	22

*NHG* Nottingham histological grade, *PR* Progesterone receptor, *Ki67* Proliferative fraction, *GPER* G protein-coupled estrogen receptor, *PM* plasma membrane, *AR* Androgen receptor

Positive associations were observed between GPER and both AIB1 (*P* = 0.01) and AR (*P* = 0.05). The evidence for a positive association between AIB1 and AR was weaker (*P* = 0.32). The associations between these three factors and other prognostic factors were also in general weak with strongest evidence for a positive association between AIB1 and Ki67 (*P* = 0.002) and for a negative association between AR expression and grade (*P* < 0.001) (Table 3).

**Table 3:** Association between AR, AIB1, GPER, and other prognostic factors^a^

	*N*	AIB1 low	AIB1 high	*P*	*N*	GPER 0	GPER 1	GPER 2 + 3 + 4	*P*	*N*	AR negative	AR positive	*P*
Nodal status													
0	122	114 (62)	8 (57)	0.56	120	32 (57)	48 (59)	40 (66)	0.43	114	6 (50)	108 (62)	0.27
1–3	35	33 (18)	2 (14)		36	11 (20)	16 (20)	9 (15)		36	2 (17)	34 (19)	
4+	41	37 (20)	4 (29)		43	13 (23)	18 (22)	12 (20)		37	4 (33)	33 (19)	
N0	122	114 (62)	8 (57)	0.72	120	32 (57)	48 (59)	40 (66)	0.35	114	6 (50)	108 (62)	0.42
*N*+	76	70 (38)	6 (43)		79	24 (43)	34 (41)	21 (34)		73	6 (50)	67 (38)	
Size													
0–20 mm	116	106 (55)	10 (77)	0.13	116	28 (47)	56 (66)	32 (52)	0.67	107	4 (31)	103 (57)	0.06
> 20 mm	89	86 (45)	3 (23)		90	31 (53)	29 (34)	30 (48)		86	9 (69)	77 (43)	
Menopause													
Pre	50	47 (25)	3 (21)		50	17 (29)	22 (27)	11 (17)		45	3 (23)	42 (24)	
Post	151	140 (75)	11 (79)	0.10	152	41 (71)	59 (73)	52 (83)	0.13	145	10 (77)	135 (76)	0.96
NHG													
1	23	21 (12)	2 (15)	0.39	24	5 (9)	14 (18)	5 (9)	0.81	23	0 (0)	23 (14)	< 0.001
2	152	141 (81)	11 (85)		152	45 (83)	61 (77)	46 (82)		142	7 (58)	135 (81)	
3	13	13 (7)	0		13	4 (7)	4 (5)	5 (9)		13	5 (42)	8 (5)	
1 + 2	175	162 (93)	13 (100)	0.31	176	50 (93)	75 (95)	51 (91)	0.75	165	7 (58)	158 (95)	< 0.001
3	13	13 (7)	0		13	4 (7)	4 (5)	5 (9)		13	5 (42)	8 (5)	
PR													
< 1%	34	31 (17)	3 (21)	0.68	37	11 (20)	14 (17)	12 (20)	0.95	35	2 (15)	33 (19)	0.76
≥ 1%	161	150 (83)	11 (79)		161	44 (80)	70 (83)	47 (80)		154	11 (85)	143 (81)	
Ki67													
Low	180	170 (94)	10 (71)	0.002	182	53 (96)	76 (92)	53 (90)	0.19	176	11 (85)	165 (93)	0.25
High	15	11 (6)	4 (29)		15	2 (4)	7 (8)	6 (10)		14	2 (15)	12 (7)	
AIB1													
Low					190	57 (100)	79 (92)	54 (89)	0.01	13	0 (0)	13 (7)	0.32
High					14	0	7 (8)	7 (11)		180	13 (100)	167 (93)	
GPER													
0										57	8 (62)	49 (27)	0.05
1										77	2 (15)	75 (41)	
2 + 3 + 4										61	3 (23)	58 (32)	

^a^For 2-by-2-tables, *P* values were calculated using Pearson’s chi2 test. For larger tables, where at least one variable is ordinal with > 2 categories, a trend version of this test was used. The latter test is equivalent to a test for zero slope in a linear regression model

### Breast cancer mortality

#### Univariable analyses

**Fig. 3:**
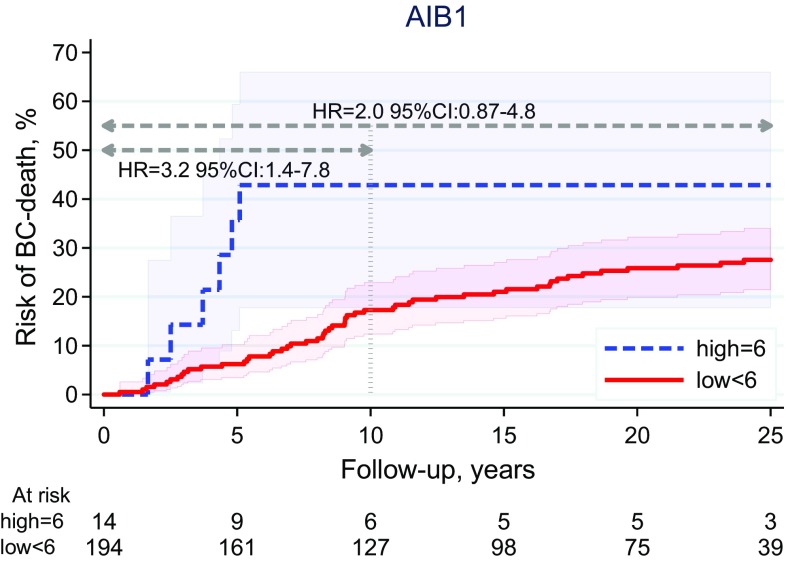
AIB1: Breast cancer mortality (10- and 25-year FU)

AIB1 (high vs. low) was associated with BCM with 10-year FU (HR 3.2, 95% CI 1.4–7.8, *P* = 0.008), but the effect and the evidence was weaker when analyzed with 25-year FU (HR 2.0, 95% CI 0.87–4.8, *P* = 0.10) (Fig. 3; Table 4). AR (positive vs. negative) showed a trend for a prognostic difference in BCM with 10-year FU (HR 0.56, 95% CI 0.17–1.8), but the evidence was very weak (*P* = 0.33), and the effect was lost when analyzed with 25-year FU (HR 0.93, 95% CI 0.29-3.0, *P* = 0.90) (Table 4; Online Resource 1). Total GPER (log-rank test for trend over the three observed categories) was neither associated with BCM with 10-year (*P* = 0.33) nor with 25-year FU (*P* = 0.55). (Table 4; Online Resource 1) Ki67 (high vs. low) and NHG (3 vs. 1 + 2) were prognostic for BCM with 10- and 25-year FU, whereas PR (positive vs. negative) was not (Table 4).

**Table 4:** Univariable analysis of breast cancer mortality (10- and 25-year FU) in invasive lobular carcinoma

	*N*	10-year FU			25-year FU		
		HR	95% CI	*P* value	HR	95% CI	*P* value
Age (years)	224	0.95	0.93–0.98	0.001	0.97	0.95–0.99	0.01
Tumor size (mm)							
≤ 20	125	1.0			1.0		
> 20	96	4.1	2.1–7.8	< 0.001	3.5	2.1–5.7	< 0.001
Nodal status (*P* < 0.001)^a^							
0+	129	1.0			1.0		
1–3+	40	1.7	0.74–4.1	0.20	1.2	0.62–2.5	0.54
> 3+	44	5.6	2.0–11	< 0.001	3.5	2.0–6.0	< 0.001
Histological grade							
1 + 2	189	1.0			1.0		
3	13	4.1	1.6–10	0.003	3.8	1.6–8.8	0.002
PR							
+ (≥ 1%)	162	1.0		0.29	1.0		
− (< 1%)	37	1.5	0.71–3.2		1.4	0.71–2.6	0.33
Ki67							
0–23%	182	1.0			1.0		
≥ 24%	15	4.9	2.2–11	<0.001	5.9	3.1–11	<0.001
AIB1							
Low + medium (score < 6)	194	1.0			1.0		
High (score 6)	14	3.2	1.4–7.8	0.008	2.0	0.87–4.8	0.10
GPER (*P* = 0.38)^a^							
Negative (0)	59	1.0			1.0		
Very weak (1)	87	0.79	0.38–1.6	0.54	0.86	0.47–1.6	0.62
Weak + moderate + strong (2–4)	63	0.69	0.300–1.6	0.38	0.87	0.45–1.7	0.69
AR							
+ (> 10%)	183	1.0			1.0		
− (≤ 10%)	13	1.8	0.56–5.9	0.33	1.1	0.34–3.5	0.90

^a^Log-rank test for trend

Sixty-five percent (125/193) of the evaluable tumors were classified as luminal A-like and the remaining as luminal B-like and the evidence for higher BCM in luminal B-like tumors was strong (10-year FU: HR 1.9, 95% CI 1.1–3.1, *P* = 0.01; 25-year FU: HR 1.9, 95% CI, 1.3–2.6 *P* < 0.001) (Online Resource 1).

#### Multivariable analysis

In a multivariable analysis adjusted for age, tumor size, nodal status, NHG, Ki67, luminal-like classification, and adjuvant systemic therapy (endocrine +/- chemo), AIB1 was associated with BCM with 10-year FU (HR 6.8, 95% CI 2.3–20, *P* = 0.001). However, with longer follow-up, the independent AIB1 effect was found to level off (25-year FU: HR 3.0, 95% CI 1.1–7.8, *P* = 0.03).

### Analyses of gene expression data

**Fig. 4:**
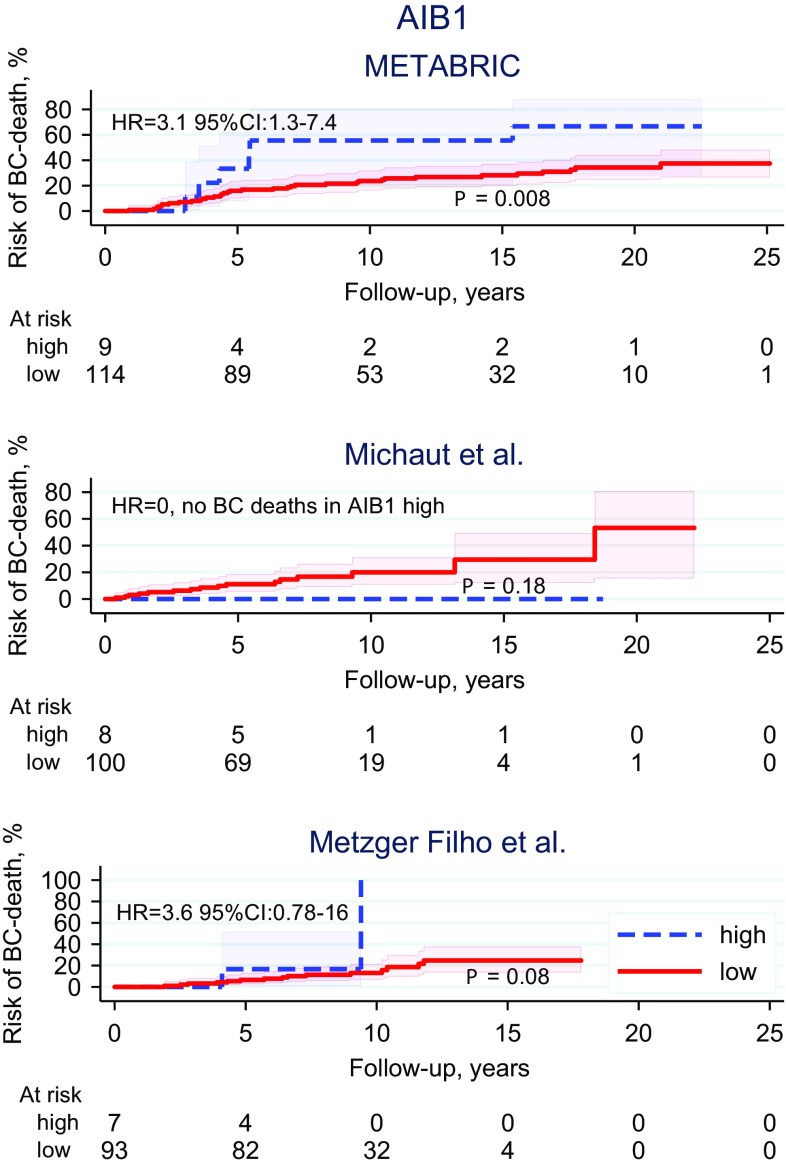
AIB1: Risk of breast cancer death assessed by gene expression data in three independent publicly available datasets of invasive lobular carcinoma (*P* value: log-rank test)

High AIB1 expression was associated with worse outcome (HR > > 1.00) in two out of the three datasets (METABRIC (HR 3.1, 95% CI 1.3–7.4, *P* = 0.01), Metzger Filho et al. (HR 3.6, 95% CI 0.78-16, *P* = 0.10)) (Fig. 4). High AR expression was associated with better outcome (HR < < 1.00) in two out of three datasets (Metzger Filho et al. (HR 0.24, 95% CI 0.07–0.87, *P* = 0.03), Michaut et al. (HR 0.35, 95% CI 0.08–1.6, *P* = 0.18)) (Online Resource 2). GPER was not associated with survival in any of the datasets (Online Resource 3).

## Discussion

In this well-characterized case series of patients with ER-positive/HER2-negative ILC, a small subgroup of 14 patients (7%) was found to have high expression of the estrogen receptor coactivator AIB1, and five of them died from breast cancer within approximately 5 years, translating to a high cumulative 5-year mortality in this subgroup compared to that in the large subgroup of patients with lower, or no, expression of AIB1 (Fig. 2). However, no late breast cancer deaths were registered in this group with six patients surviving more than 10 years and three more than 25 years. Hence, the estimated mortality ratio for AIB1 (high vs. low) was strongly dependent on FU time. In univariable analysis, it was estimated to 3.2 and 2.0 with FU of 10 and 25 years, respectively. Furthermore, the uncertainty in the estimated cumulative BCM for high-AIB1 is large for this group compared to low-AIB1, as reflected by the shaded 95% pointwise confidence bands in Fig. 2. Nevertheless, with 10-year FU, AIB1 was found to be an independent prognostic factor for BCM after adjustment for age, tumor size, nodal status, NHG, Ki67, luminal-like classification, and adjuvant systemic therapy.

Forty-one percent developed a loco-, regional-, or distant recurrence, as compared to 29% BC deaths from BC. Considering this, a complementary analysis was made, exploring the association between AIB1 and recurrence, and essentially the same prognostic effect of AIB1 was seen for the endpoint recurrence-free interval [43] (data not shown).

The association between high-AIB1 and poor prognosis is in agreement with previous BC studies from our group (including NST) [23, 24, 27], but the percentage of tumors with high-AIB1 was found to be lower in this ILC cohort. Results from an exploratory analysis with further subdivision of AIB1 into low < 5 (*N* = 98), intermediate 5-5.5 (*N* = 96), and high 6 (*N* = 14), were in concert with previous studies from our group [24, 27], and showed no difference in clinical outcome between the low and intermediate groups (data not shown). These findings support the choice of score 6 as an appropriate cut-off value in the present study. Further larger studies are warranted in order to find the most optimal cut-off for AIB1 in ER-positive/HER2-negative ILC.

In line with other studies, AIB1 was also associated with high Ki67 [27] but in contrast, AIB1 was not associated with NHG 3. As expected, only a small fraction of the ILCs were classified as NHG 3 (*N* = 13), and furthermore, none of them had high AIB1 expression.

In addition to AIB1, we studied the prognostic importance of AR and GPER, but without finding any significant results associated with outcome for these two endocrine biomarkers. The skewed distribution of both AR (only 7% AR negative) and GPER (only 1% of the tumors showed a moderate/strong total GPER staining and only 2% were GPER positive in the plasma membrane) reduces the power to detect prognostic effects.

The low fraction of tumors, with a high total GPER/PM GPER positivity in ILC, are in contrast to previous results on ER-positive BC (including NST) from our group [29, 30], and may indicate a subtype-specific difference compared to NST.

Results from analyses of the publicly available gene expression ILC datasets strengthened our IHC findings for AIB1. High expression of AIB1 was a negative prognostic factor in two out of three datasets (HR 3.1 and HR 3.6, respectively). The evidence for association between high-AIB1 and worse outcome in these datasets was, however, modest (*P* = 0.01 and *P* = 0.10, respectively).

As expected, we also found that Ki67, NHG, and the luminal-like classification were prognostic, and the same trends could be identified in the gene expression ILC datasets (data not shown).

One of the strengths of the present study is the reevaluation of histological type by clinical pathologists specialized in breast pathology. Another is the long FU time (median 26 years), since the lobular subtype, with a high proportion of luminal A-like tumors, are associated with an increased risk of late recurrences (e.g., in this cohort, 21 out of 66 breast cancer deaths occurred ≥ 10 years after diagnosis).

The study also has limitations. Besides the limited number of patients and the skewed distribution of the experimental biomarkers, one might also argue that TMAs are not optimal for IHC evaluation of biomarkers in ILC, with a scattered growth pattern characterized by single-file infiltrating cells. However, when comparing the present results for ER, PR, and Ki67, analyzed on TMA, with the previous study from our group, using whole tissue sections instead, essentially the same results were obtained for prognostic considerations.

The majority of the patients (58%) in this study did not receive any adjuvant systemic therapy. Most of the patients with endocrine treatment received tamoxifen for 2 years and only 2% received chemotherapy. Furthermore, if adjuvant systemic therapy (endocrine +/- chemo) had been given in accordance with current treatment guidelines, the BCM in this ILC cohort would probably have been lower, and some of the very late recurrences (≥ 10 year past diagnosis) could potentially have been avoided.

In conclusion, this retrospective study shows prognostic value of AIB1 in ER-positive/HER2-negative ILC both when assessed by immunohistochemistry and by gene expression assays. Validation of this finding in independent cohorts is warranted.

## Electronic supplementary material

Below is the link to the electronic supplementary material.

Supplementary material 1 (PDF 482 KB)

## Footnotes

1 When averaging over two scores differing by one unit, the result will be non-integer.
