# Supplementary material for: The estrogen receptor coactivator AIB1 is a new putative prognostic biomarker in ER-positive/HER2-negative invasive lobular carcinoma of the breast
Source: Breast Cancer Res Treat. 2019 Feb 22;175(2):305–16. doi: 10.1007/s10549-019-05138-7 (PMC6533234; doi:10.1007/s10549-019-05138-7)
Supplement: Supplementary file 1 — Supplementary material 1 (PDF 482 KB) [file 10549_2019_5138_MOESM1_ESM.pdf]

Supplementary material

Title: The estrogen receptor coactivator AIB1 is a new putative prognostic biomarker in ER-positive/HER2-negative invasive lobular carcinoma

Journal: Breast Cancer Research and Treatment

Authors: Ulrik Narbe, Martin Sjöström, Carina Forsare, Pär-Ola Bendahl, Sara Alkner, L.M. Fredrik Leeb-Lundberg, Kristina Lövgren, Lisa Rydén, Christian Ingvar and Mårten Fernö

Corresponding author: Ulrik Narbe, ulrik.narbe@med.lu.se, Department of Clinical Sciences, Division of Oncology and Pathology, Medicon Village, SE-223 81 Lund, Lund University, Sweden

Online Resource 1

A

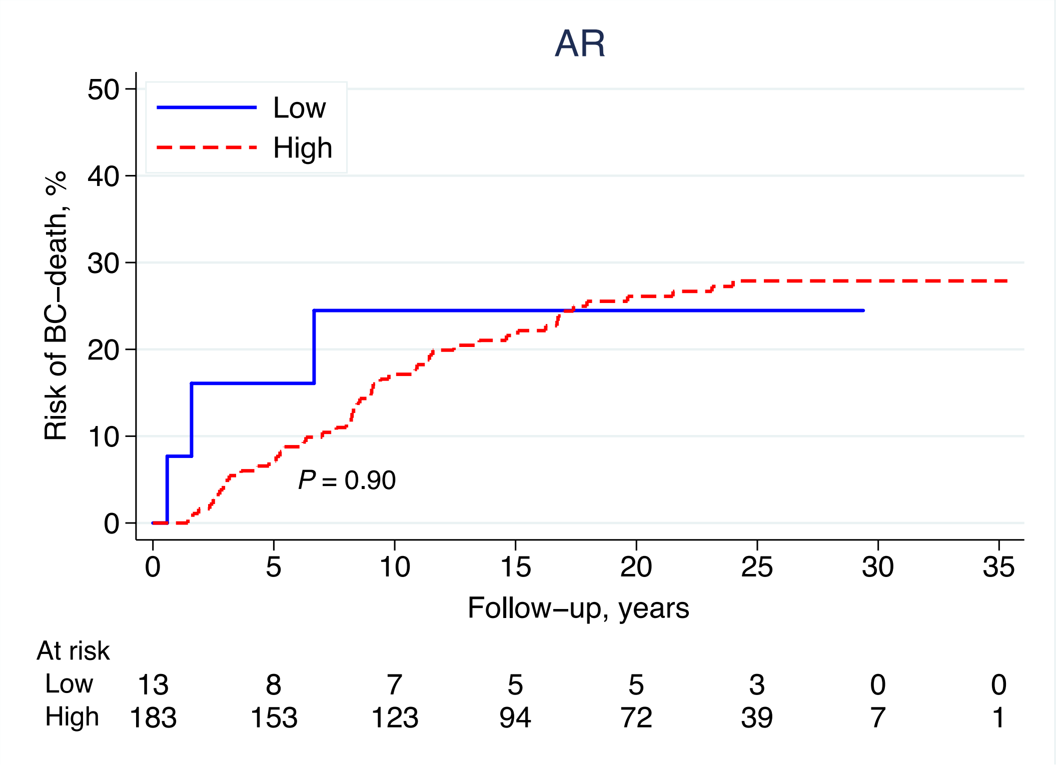

B

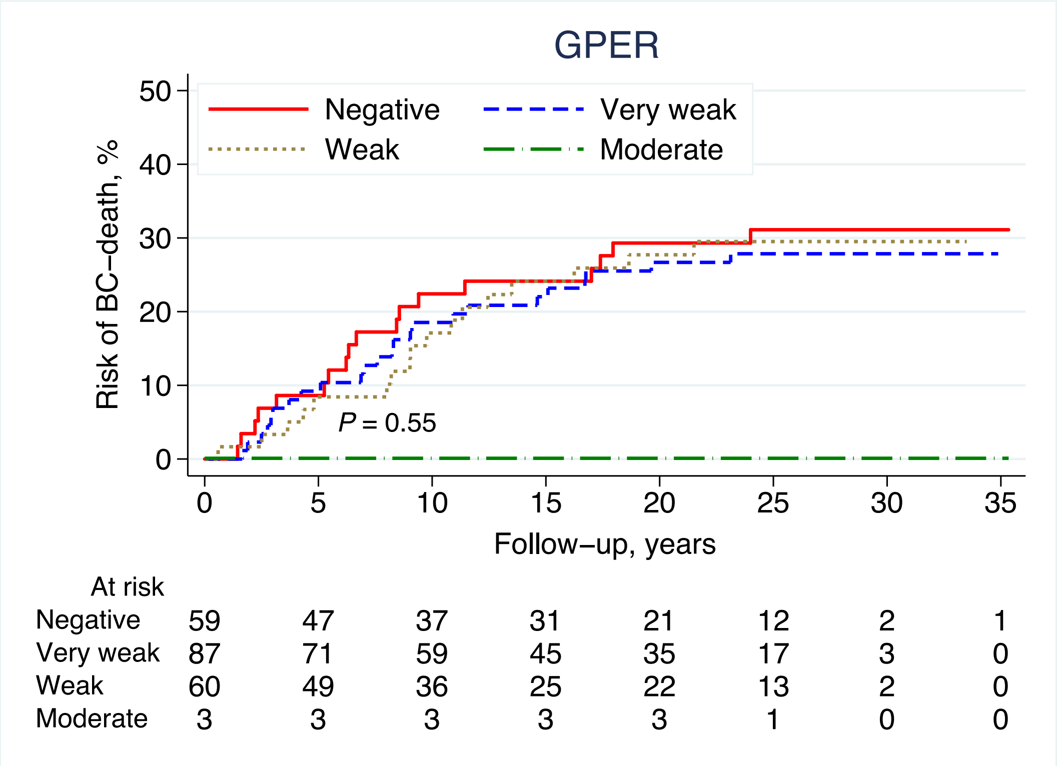
GPER
Negative
Very weak
Weak
Moderate
Risk of BC−death, %
50
40
30
20
10
0
P = 0.55
0
5
10
15
20
25
30
35
Follow−up, years
At risk
Negative 59 47 37 31 21 12 2 1
Very weak 87 71 59 45 35 17 3 0
Weak 60 49 36 25 22 13 2 0
Moderate 3 3 3 3 3 1 0 0

C

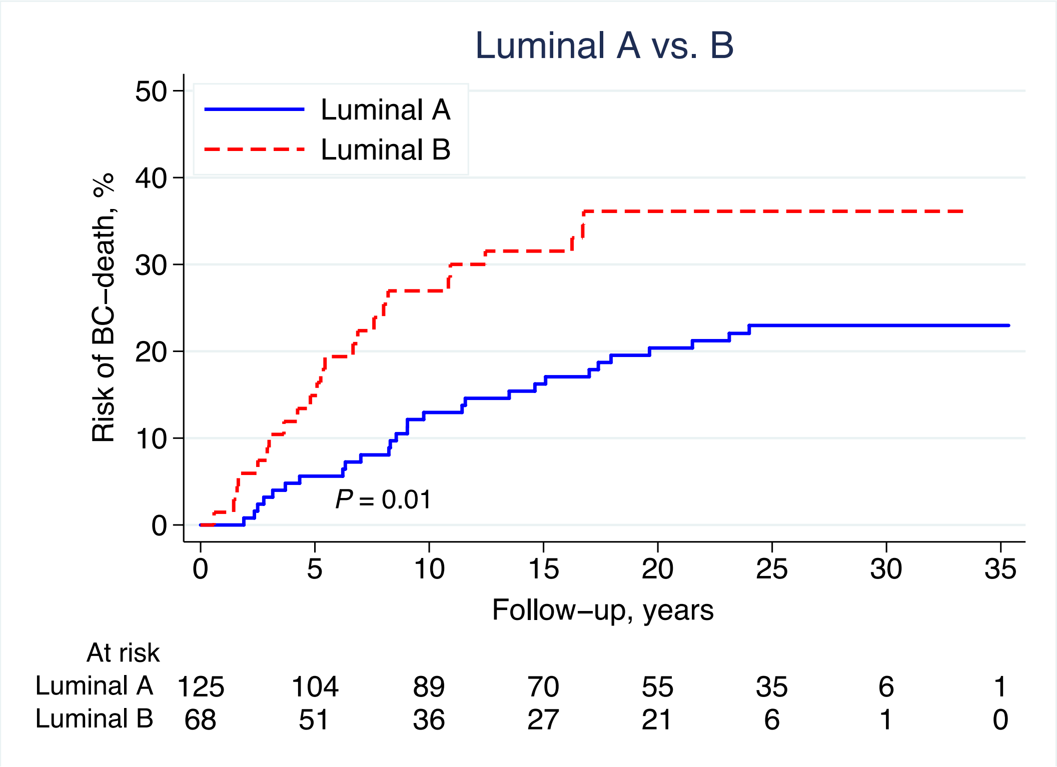
Luminal A vs. B
Luminal A
Luminal B
Risk of BC−death, %
50
40
30
20
10
0
P = 0.01
0
5
10
15
20
25
30
35
Follow−up, years
At risk
Luminal A 125 104 89 70 55 35 6 1
Luminal B 68 51 36 27 21 6 1 0

Online Resource 2

A

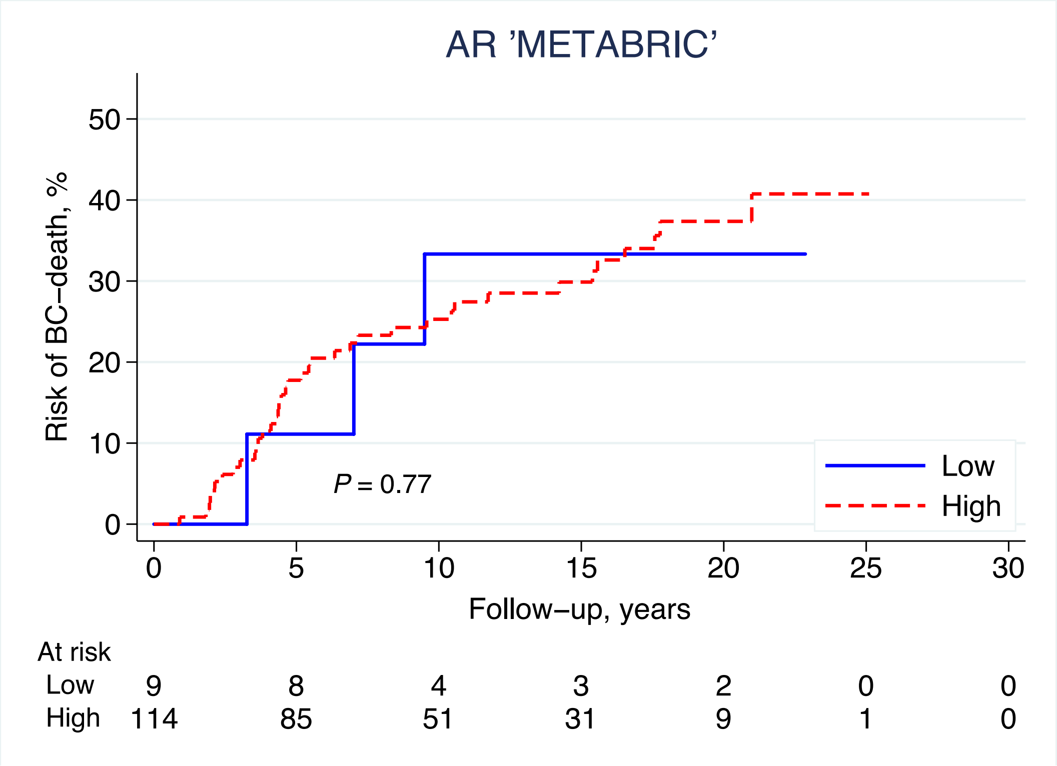

B

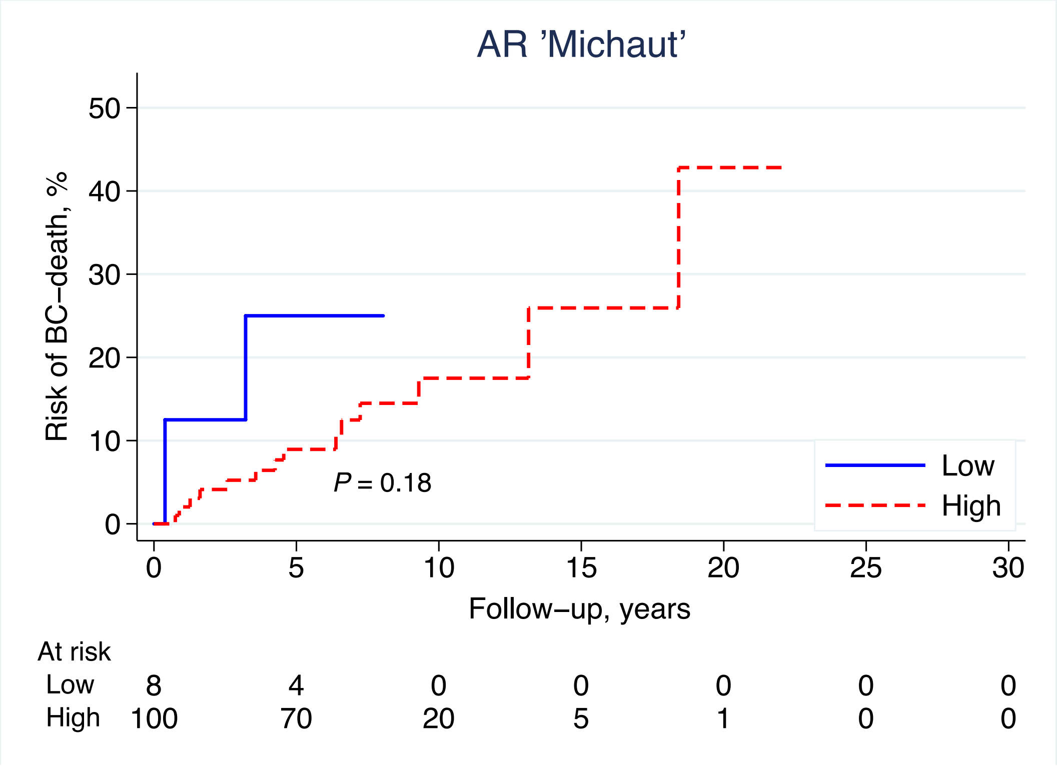

C

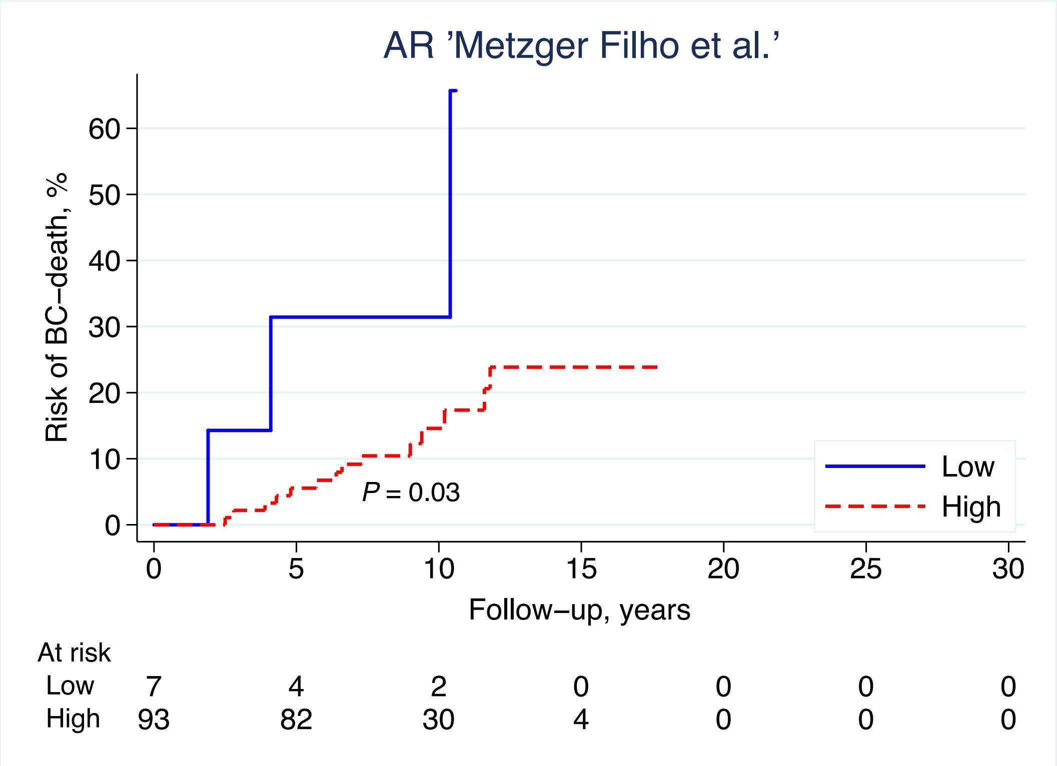

AR 'Metzger Filho et al.'
Risk of BC−death, %
60
50
40
30
20
10
0
P = 0.03
Low
High
0
5
10
15
20
25
30
Follow−up, years
At risk
Low 7 4 2 0 0 0 0
High 93 82 30 4 0 0 0

Online Resource 3

A

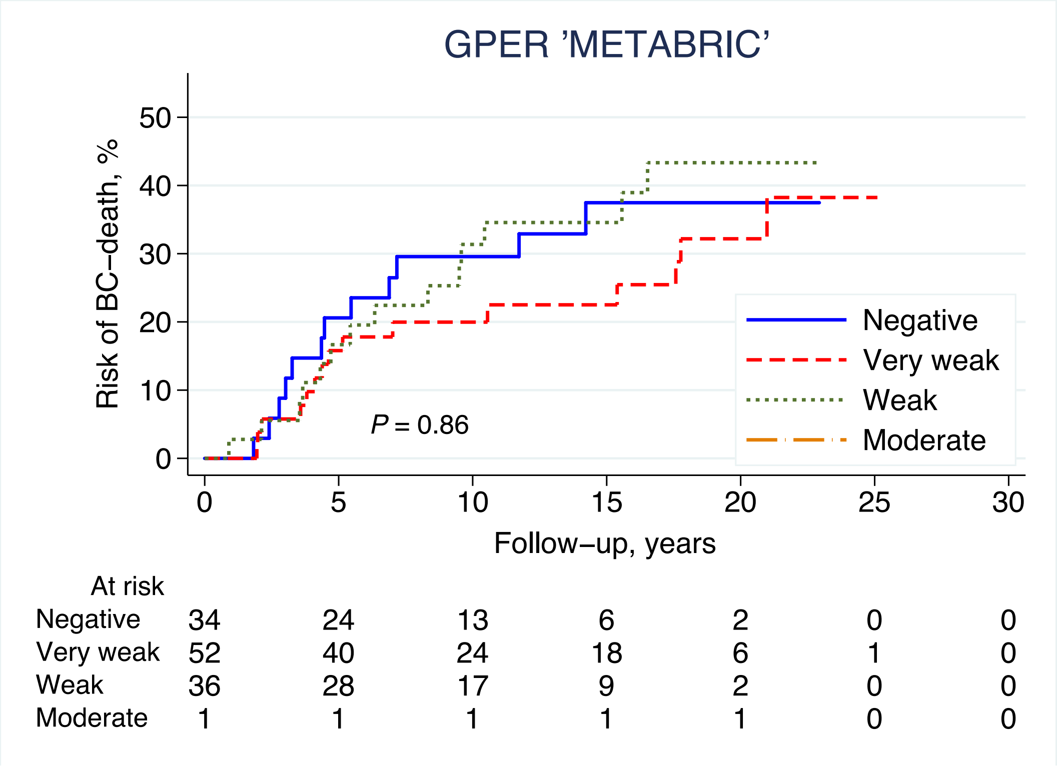

B

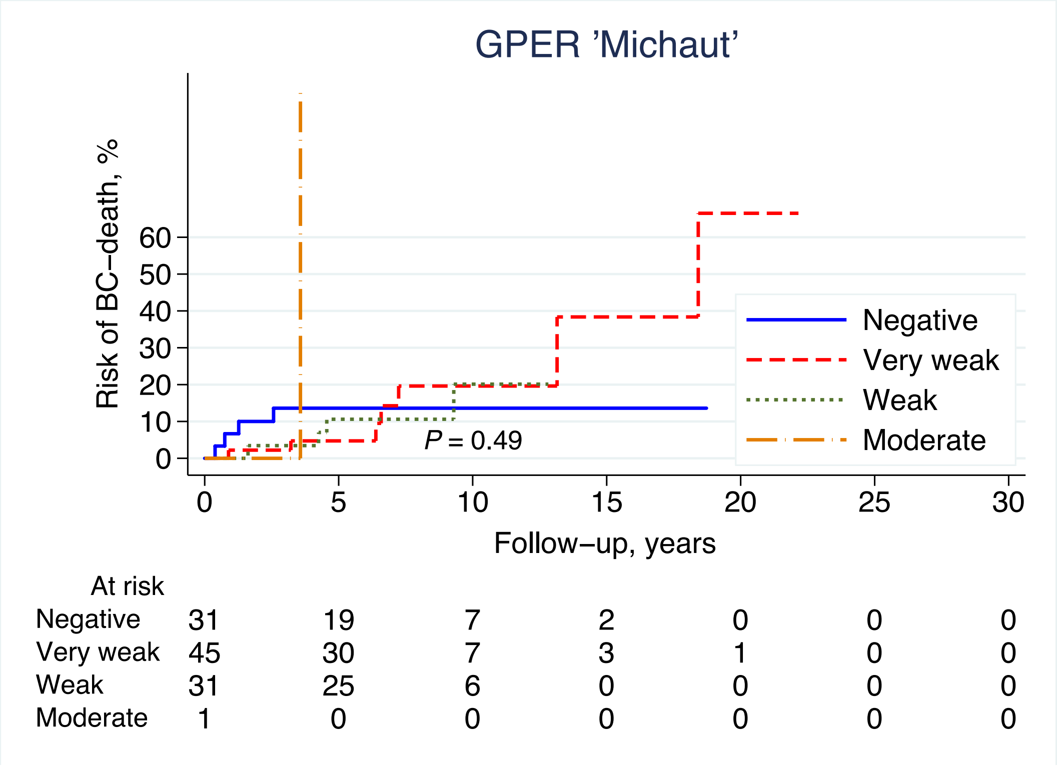

C

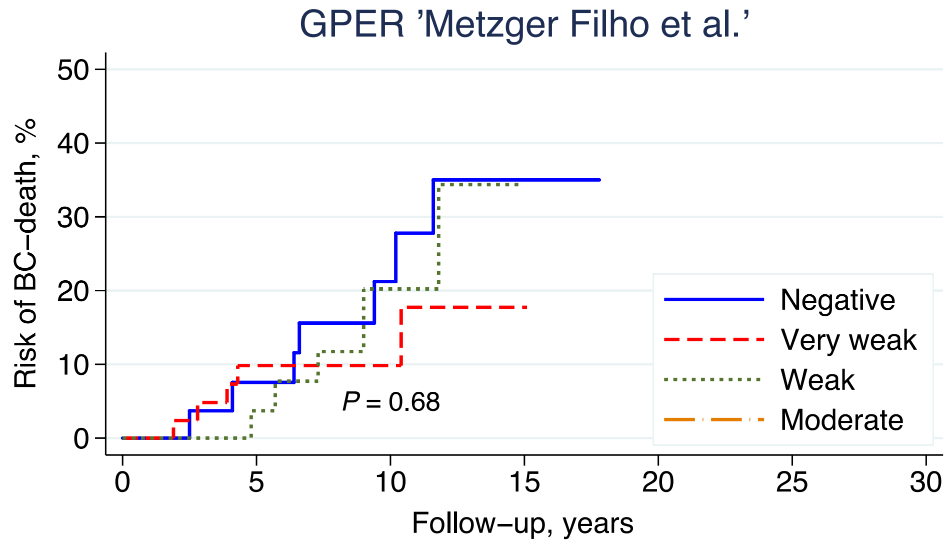

At risk							
Negative	28	24	13	2	0	0	0
Very weak	42	35	13	2	0	0	0
Weak	29	26	6	0	0	0	0
Moderate	1	1	0	0	0	0	0
